# Findings regarding non-sexual penile fracture in a referral emergency hospital

**DOI:** 10.1590/S1677-5538.IBJU.2020.0420

**Published:** 2021-02-03

**Authors:** Rodrigo Barros, Alex Schul, Andre G. Cavalcanti, Luciano Alves Favorito, Leandro Koifman

**Affiliations:** 1 Hospital Municipal Souza Aguiar Departamento de Urologia Rio de Janeiro RJ Brasil Departamento de Urologia do Hospital Municipal Souza Aguiar, Rio de Janeiro, RJ, Brasil; 2 Universidade Federal Fluminense - UFF Niterói RJ Brasil Universidade Federal Fluminense - UFF, Niterói, RJ, Brasil; 3 Universidade Federal do Estado do Rio de Janeiro - UNIRIO RJ Brasil Universidade Federal do Estado do Rio de Janeiro - UNIRIO, RJ, Brasil; 4 Universidade do Estado do Rio de Janeiro - UERJ Rio de Janeiro RJ Brasil Universidade do Estado do Rio de Janeiro - UERJ, RJ, Rio de Janeiro, Brasil

**Keywords:** Penis, Emergency Service, Hospital, Fractures

## Abstract

**Purpose:**

To describe penile fracture (PF) findings with non-sexual etiology in a referral emergency hospital, with emphasis on demographic data, clinical and intraoperative findings and long-term outcomes.

**Materials and Methods:**

Patients with PF of non-sexual cause operated at our institution from January 2014 to January 2019 were submitted to surgical treatment and monitored for at least three months after surgery. Etiology of trauma, epidemiological and clinical presentation data, time to intervention and operative findings were reviewed retrospectively. The evaluation of postoperative erectile function was carried out by filling out the International Index of Erection Function - 5 (IIEF-5). The tool used to assess urinary function was the International Prostate Symptom Score (IPSS) questionnaire.

**Results:**

Of a total of 149 patients submitted to surgical treatment for PF, 18 (12%) reported non-sexual etiology. Twelve (66.6%) cases were due to penile manipulation through the act of bending the penis during morning erection, three (16.6%) when rolling over in bed with erect penis, one (5.5%) when embracing the wife during erection, one (5.5%) to laying on the partner with erect penis and the other (5.5%) when sitting on the toilet with an erection. Operative findings were unilateral corpus cavernosum injury in all cases. Only one (5.5%) patient had a partial urethral lesion. Follow-up time varied from 3 to 18 months (mean, 10.1 months). Three (16.6%) patients developed erectile dysfunction six months after surgery. However, all of them responded to treatment with IPDE-5 and reported improvement of erection, with no need for medication, on reevaluation after 18 months. One (5.5%) patient developed penile curvature < 30 degrees. Thirteen (72.2%) patients developed penile nodules. No patient presented voiding complaints during follow-up.

**Conclusions:**

PF is a rare urologic emergency, especially with the non-sexual etiology. However, PF should always be considered when the clinical presentation is suggestive, regardless of the etiology. Penile manipulation and roll over in bed were the most common non-sexual causes. These cases are related to low-energy traumas, usually leading to unilateral rupture of corpus cavernosum. Urethral involvement is uncommon but may be present. Early treatment has good long-term clinical outcome, especially when performed in specialized centers with extensive experience in FP.

## INTRODUCTION

Penile fracture (PF) is a relatively uncommon injury and generally under-reported or hidden due fear and embarrassment of the patient (1). Various authors have suggested the existence of geographical variation in the etiology of PF. This may be related to the religious, cultural and behavioral habits of each region. Sexual etiology, such as vigorous intercourse and masturbation, are the main causes of PF in Western countries (2). In some Middle Eastern countries, non-sexual etiology is the most common cause of PF. As mechanisms of non-sexual injury we can include the rolling over in bed with nocturnal erection and taqaandan (3, 4). Taqaandan is a self-inflicted injury, common in Iran, consisting of intentional forceful acute bending of part of the shaft of the erect penis in a downward, upward, or lateral direction while holding the other part stationary, to cause detumescence of the penis, often to release tension (4). Other more rare causes reported include trauma to the penis while on the toilet or on a bed column (5).

Usually, patients report a snapping sound, followed by immediate pain, penile detumescence and hematoma. The classic presentation of PF is enough for exploration (1). Surgical exploration can demonstrate unilateral or bilateral lesion of corpus cavernosum. The presence of urethral injury is usually associated with high-energy trauma resulting in bilateral corpora cavernosa involvement (2).

The aim of this study is to describe PF findings with non-sexual cause in a referral emergency urological institution, with emphasis on demographic data, clinical and intraoperative findings and long-term outcomes.

## MATERIAL AND METHODS

Patients diagnosed with PF of non-sexual cause operated at our institution from January 2014 to January 2019 were included in this study. Etiology of trauma, epidemiological and clinical presentation data, time to intervention and operative findings were reviewed retrospectively using the PF database of the andrology outpatient sector of our hospital. Our institution is the biggest public urologic emergency unit in Rio de Janeiro, Brazil.

All patients were submitted to surgical treatment after diagnosis, independent of the time between trauma and hospital admission. Surgery was always performed by one of the experienced urologists of our emergency team, accompanied by one of our residents. The technique used is standardized in our institution and consists of making a circular sub-coronal incision and degloving the penis, with wide exposure of the corpus cavernosum and urethra. The hematoma was evacuated, corpus cavernosum injury was identified, surgical debridement was performed and the tunica albuginea was sutured using simple interrupted 3-0 polyglactin sutures. Partial urethral lesions were treated with simple interrupted 5-0 polyglactin sutures over a Foley catheter. In cases of total urethral lesion, the urethral edges were dissected at both sides, spatulated and closed with interrupted sutures using 5-0 polyglactin. The urethral catheter was left in place for 10-14 days in cases of partial injury and for 14-21 days in cases of complete urethral lesions (6).

Patients were monitored for at least three months after surgery. They were examined and interviewed about any sexual dysfunction. The evaluation of postoperative erectile function was carried out by filling out the International Index of Erection Function - 5 (IIEF-5) 6, 12 and 18 months after surgery. The tool used to assess urinary function was the International Prostate Symptom Score (IPSS) questionnaire three months after surgery.

The study protocol was approved by the ethics and human research committee of the institution.

## RESULTS

Of a total of 149 patients submitted to surgical treatment for PF between January 2014 and January 2019, 18 (12%) reported non-sexual cause. Twelve (66.6%) cases were due to penile manipulation through the act of bending the penis during morning erection, three (16.6%) when rolling over in bed with erect penis, one (5.5%) when embracing the wife during erection, one (5.5%) to laying on the partner with erect penis and the other (5.5%) when sitting on the toilet with an erection (Table-1).

**Table 1:** Non-sexual etiology of penile fracture.

Mechanisms of trauma	Cases (%)
Penile manipulation	12 (66.6)
Roll over in bed	03 (16.6)
Sitting on the toilet with erect penis	01 (5.5)
Embracing the partner with the erect penis	01 (5.5)
Lying on the partner with the erect penis	01 (5.5)

**Figure 1:**
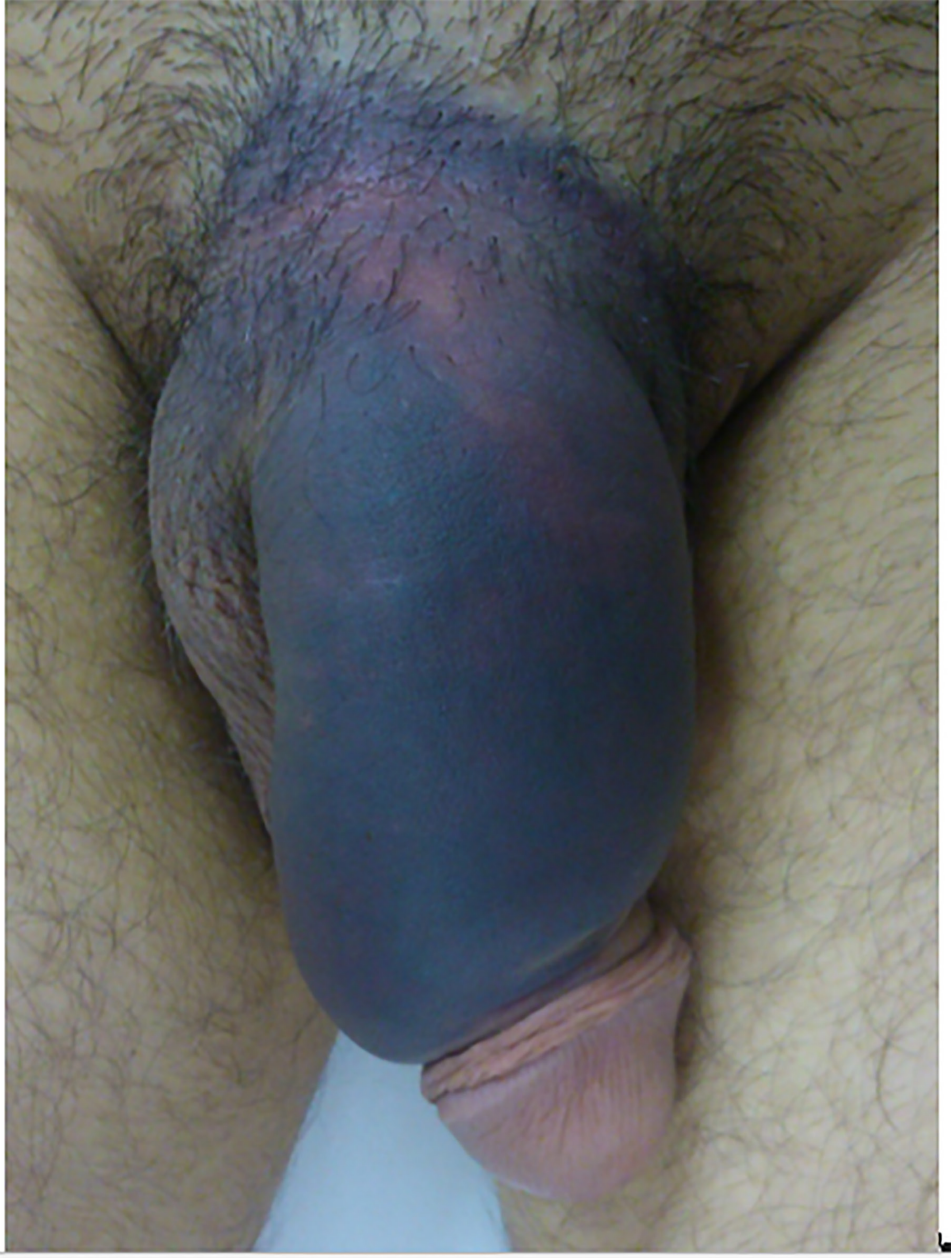
Penile hematoma in patient presenting penile fracture after rolling over in bed.

Age ranged from 24 to 69 years (mean=41.6). Ten (55.5%) patients were single and eight (44.5%) were married. Regarding clinical presentation, the main finding was hematoma (Figure-1), observed in all patients, followed by cracking sound in 17 (94.4%) cases, immediate penile detumescence in 15 (83.3%) and pain in 14 (77.7%). Urethral bleeding, hematuria and urinary retention were not observed in any case. Diagnosis was mainly based on history and physical examination, using ultrasound (US) in three (16.6%) doubtful cases for confirmation.

**Figure 2:**
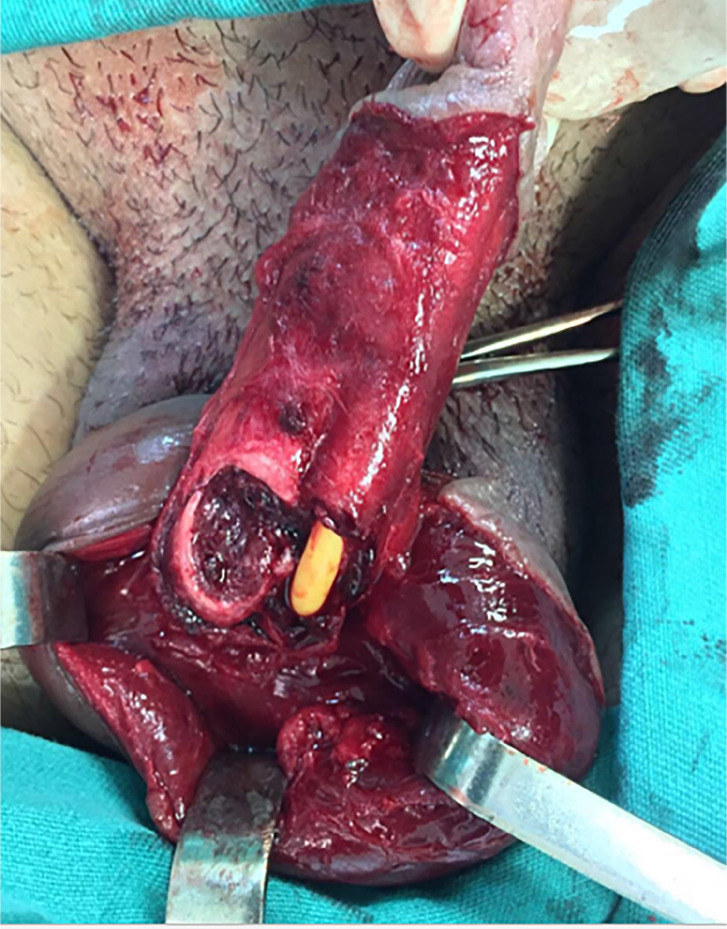
Unilateral rupture of right corpus cavernosum, with partial urethral lesion in the only patient with urethral involvement.

Operative findings were unilateral corpus cavernosum injury in all cases. Only one (5.5%) patient had a partial urethral lesion (Figure-2). Time between trauma and surgery varied from 3 to 168 hours (mean, 36.7 hours).

Follow-up time varied from 3 to 18 months (mean 10.1 months). Regarding sexual complications, three (16.6%) patients developed erectile dysfunction (ED) after six months, mild in two cases and mild to moderate in one according to IIEF-5. All patients reported having normal erectile function without any type of drug treatment at the time of the trauma. Two of them were under 50 years old, without comorbidities, and the other was 66 years old and was an ex-smoker. All patients responded to treatment with phosphodiesterase type 5 inhibitor (IPDE-5) and were monitored for the evolution of erectile function. Two patients reported improvement of erection, with no need for medication, on reevaluation after 12 months and one after 18 months. One (5.5%) patient developed penile curvature <30 degrees, with no need for surgical correction. Thirteen (72.2%) patients developed penile nodules, all at the same site of the surgical repair on the tunica. Table-2 shows the demographics, clinical findings and sexual complications. Based on the IPSS questions, no patient presented voiding complaints during follow-up. Although it is difficult to adequately administer the IPSS questionnaire in emergency situations, no patient reported urinary complaints before the trauma.

**Table 2:** Demographics, clinical findings and sexual complications.

Case	Age (years)	Marital status	Time of presentation (hours)	Etiology	Hematoma	Cracking sound	Detumescence	Pain	Urethra lbleeding	Sexual Complications
1	55	Married	10	Sitting on the toilet	Yes	Yes	Yes	Yes	No	Nodule
2	39	Married	8	Embracing the partner	Yes	Yes	Yes	Yes	No	Nodule
3	30	Single	3	Penile manipulation	Yes	Yes	Yes	No	No	No
4	32	Single	7	Penile manipulation	Yes	Yes	Yes	Yes	No	Nodule
5	52	Single	26	Penile manipulation	Yes	Yes	Yes	Yes	No	Nodule and Penile Curvature
6	29	Married	24	Penile manipulation	Yes	Yes	Yes	Yes	No	Erectile Dysfunction
7	66	Married	84	Lying on the partner	Yes	Yes	Yes	No	No	Nodule and Erectile Dysfunction
8	46	Silgle	6	Penile manipulation	Yes	Yes	Yes	Yes	No	Erectile Dysfunction
9	30	Single	13	Penile manipulation	Yes	Yes	No	No	No	Nodule
10	32	Single	12	Penile manipulation	Yes	Yes	Yes	Yes	No	Nodule
11	36	Married	48	*Roll in bed*	Yes	No	No	Yes	No	Nodule
12	30	Single	3	Penile manipulation	Yes	Yes	Yes	No	No	No
13	39	Single	12	*Roll in bed*	Yes	Yes	No	Yes	No	Nodule
14	24	Single	144	Penile manipulation	Yes	Yes	Yes	Yes	No	Nodule
15	69	Married	168	Penile manipulation	Yes	Yes	Yes	Yes	No	Nodule
16	43	Single	48	Penile manipulation	Yes	Yes	Yes	Yes	No	Nodule
17	34	Married	10	*Roll in bed*	Yes	Yes	Yes	Yes	No	Nodule
18	64	Married	36	Penile manipulation	Yes	Yes	Yes	Yes	No	Nodule

Finally, we separated the last 18 cases of PF with sexual etiology from our database in order to compare the operative findings and sexual complications with our sample of non-sexual etiology. Unilateral injuries of the corpus cavernosum were found in 12 patients (66.7%) and bilateral injuries were identified in six (33.3%) patients. Urethral injuries were observed in seven cases (38.8%), including five (27.7%) partial lesions and two (11.1%) complete lesions. Fourteen (77.7%) patients developed penile nodule, three (16.6%) patients had penile curvature <30 degrees and two (11.1%) patients developed ED. However, both reported improvement of erection, with no need for medication, on reevaluation after 18 months.

## DISCUSSION

Etiology can vary due to many factors, such as geographic region, sociocultural characteristics, marital status and masturbation habits. Heterosexual vaginal intercourse is reported as the main cause of PF in the World, and there have been reports of occurrence during homossexual anal coitus (7). In Mediterranean and Middle Eastern countries, the majority of cases result from self-inflicted penile manipulation during erection to achieve detumescence (8). In Japan, many cases are reported resulting from masturbation and rolling over in bed on to an erect penis (9). Other mechanisms of trauma occasionally reported in the literature are placing an erect penis into tight pants, striking a toilet seat and hitting a bedpost (10, 11).

Yapanoglu et al. (2009) reported results of 42 men with PF and the mechanisms described were due to straightening or bending by hand in 15 (35.7%), during sexual intercourse in 13 (30.9%), due to turning over or falling out of bed in nine (21.4%), due to slamming in a door in four cases (9.5%) and due to a horse kick in one (2.3%) (12). According to a series from Iran, the most common mechanisms of PF was taghaandan in 269 cases (76.4%), coital injury in 28 (7.9%), rolling over with erect penis in 16 (4.5%), falling on erect penis in 10 (2.8%), manipulation during sleep in 7 (1.9%), and miscellaneous causes in 21 (5.9%). Taghaandan is acute bending of the erect penis in an attempt to achieve detumescence. It is more common in unemployed and uneducated patients who do not know the properties of penile tissue and think that the penis has cartilage or do not believe that PF can result from that manipulation. The most common motives for this practice are for pleasure, to provide the penis with relaxation and relief, and to produce detumescence (13). In our study, 12% the reported cases were of non-sexual cause, with the main mechanism being penile manipulation through the act of bending the penis during morning erection in 12 (66.6%) patients, without a specific motivation. Most of them reported they were in the habit of pushing the penis down, as if it were an act of stretching, and were unaware that this could cause trauma to the penis, just as they had never heard of PF. In addition, eight (50%) patients were referred by a general practitioner, without diagnostic hypothesis of PF, perhaps due to associating PF exclusively with sexual intercourse. This demonstrates how lay people, as well as doctors who are not urologists, are unaware of the etiology, history and clinical presentation of PF.

US is a readily available and non-invasive technology that can confirm PF as a defect in the tunica albuginea with adjacent hematoma. Moreover, US can demonstrate hematoma with intact tunica albuginea in cases with rupture of a dorsal vein of the penis (14). Therefore, US can be particularly used in cases that are doubtful cases or with low suspicion of corpora cavernosum injury (2). We needed to use US in three (16.6%) doubtful cases, with less pronounced clinical presentation, probably due to low-energy traumas. Magnetic resonance imaging (MRI) is more reliable than US. MRI has high sensitivity and negative predictive value for tunical rupture or concomitant urethral lesion, so it can be applicable in doubtful cases to exclude the diagnosis of PF and avoid unnecessary surgery (6). However, we do not use MRI in any cases because we do not have this technology at our institution.

The most common intraoperative finding in PF is the unilateral lesion of the corpora cavernosa, but bilateral corporal injury was reported in 4-10% of cases (15, 16). Urethral lesions are usually associated with bilateral fractures of the corpus cavernosum due to high-energy trauma such as vigorous intercourse in the ‘man-on-top’ and ‘doggy style’ positions (17). The incidence of urethral lesion is significantly higher in the United States and Europe, ranging from 20-38%, than in Asia and the Middle East, which have a prevalence of less than 3% (13, 18). This probably occurs because the etiology varies in different geographic regions, where in the United States the majority of cases result from sexual activity and in the Mediterranean and Middle Eastern countries the main cause is penile manipulation (19). Urethral injury is more frequently associated with coital fracture, when more vigorous force is expended, than with manipulation or masturbation injury, in which lesser force is involved (4). Rahman et al. (2016) reported their experience with penile manipulation during masturbation in six of seven patients of PF and no patient had any associated urethral injury (20). In our study, we found unilateral corpus cavernosum injury in all cases, with only one patient having partial urethral tears, in which the patient reported having excessive force during penile manipulation. Compared to our sample of PF with sexual etiology, bilateral lesions of the corpus cavernosum and urethral lesions were more common in this type of etiology, probably because it is associated with a high-energy trauma.

Long-term complications following PF surgical treatment include painful erection, skin necrosis, penile nodules, penile curvature, erectile dysfunction (ED), urethrocutaneous fistula and urethral stricture (21).

In the study of Zargooshi (2009), penile fibrotic nodules were found at follow-up in 330 patients (93.7%) and of the 217 patients who had partners, only three (1.3%) were impotent (13). In our series, we found penile nodules in thirteen (72.2%) cases. Three (16.6%) patients developed ED six months after surgery. However, all of them responded to treatment with IPDE-5 and reported improvement of erection, with no need for medication, on reevaluation after 18 months. We believe these patients are affected psychologically after surgery, but usually improve with time. Penile curvature is reported to occur in about 5-14% of patients and is generally mild, not needing corrective surgery (22). In corroboration, we found only one (5.5%) case of curvature <30 degrees, where the treatment was conservative. These findings were similar to those found in our sample of sexual etiology.

Several studies have demonstrated good results of urethral reconstruction after PF (23). El-Assmy (2010) found one case of urethral narrowing in fourteen cases, which required regular dilatation for one month (24). Raheem et al. (2014) described their experience with 11 cases PF associated with complete urethral injury after immediate surgical reconstruction and only one patient developed a ring stricture at the anterior urethra, which was treated successfully by regular urethral dilatation (25). In our study, we had only one case of partial urethral injury, with restoration of normal urinary function after reconstruction.

Our results demonstrate the importance of early repair in PF, in agreement with several studies that emphasize immediate surgery in PF in order to minimize physical and psychological morbidity as well as reduce the length of inpatient stay (26).

The present study has some limitations. It is based on a small sample and part of the data were obtained retrospectively. However, to our knowledge this is the first study to specifically and exclusively address the findings and outcomes of PF with non-sexual etiology in a referral emergency hospital.

## CONCLUSION

PF is a rare urologic emergency, especially with the non-sexual etiology. However, PF should always be considered when the clinical presentation is suggestive, regardless of the etiology. Penile manipulation and roll over in bed were the most common non-sexual causes. These cases are related to low-energy traumas, usually leading to unilateral rupture of corpus cavernosum. Urethral involvement is uncommon but may be present. Early treatment has good long-term clinical outcome, especially when performed in specialized centers with extensive experience in FP.
